# Chemical Constituents and Biological Studies of the Leaves of *Grevillea robusta*

**DOI:** 10.3390/molecules16119331

**Published:** 2011-11-07

**Authors:** Ta-Hsien Chuang, Hsiu-Hui Chan, Tian-Shung Wu, Chien-Fu Li

**Affiliations:** 1 School of Pharmacy, China Medical University, Taichung 40402, Taiwan; Email: tswu@mail.ncku.edu.tw (T.-S.W.); lijeff12171@msn.com (C.-F.L.); 2 Chinese Medicinal Research and Development Center, China Medical University and Hospital, Taichung 40402, Taiwan; Email: hsiuhui.chan@gmail.com (H.-H.C.); 3 Department of Chemistry, National Cheng Kung University, Tainan 70101, Taiwan

**Keywords:** *Grevillea robusta*, proteaceae, cytotoxicity, DPPH, tyrosinase inhibition activity

## Abstract

Three new compounds: Graviquinone (**1**), *cis*-3-hydroxy-5-pentadecylcyclohexanone (**2**), and methyl 5-ethoxy-2-hydroxycinnamate (**3**), and thirty-eight known compounds were isolated and identified from the leaves of *Grevillea robusta*. The structures of these compounds were determined by spectroscopic and chemical transformation methods. Graviquinone (**1**) showed the strongest cytotoxicity against MCF-7, NCI-H460, and SF-268 cell lines. Methyl 2,5-dihydroxycinnamate (**4**), graviphane (**13**), and dehydrograviphane (**14**) exhibited very potent DPPH scavenging activity compared with α-tocopherol. Methyl 2,5-dihydroxycinnamate (**4**) and bis-norstriatol (**17**) demonstrated strong inhibition of L-DOPA oxidation by mushroom tyrosinase compared with kojic acid.

## 1. Introduction

**Figure 1:**
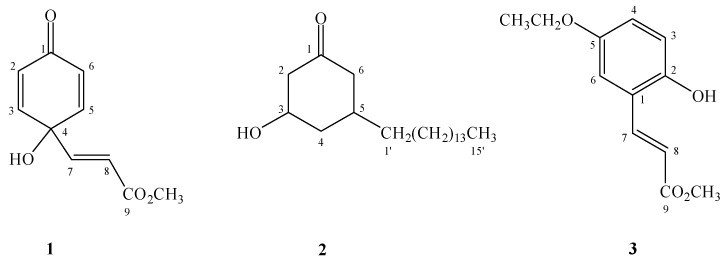
Structures of compounds **1–3**.

*Grevillea robusta* A. CUNN (Proteaceae), commonly known as “silky oak”, is native to Australia [1,2]. In Taiwan, it is cultivated as a shade tree. So far alkylresorcinols, macrocyclic phenols, and cinnamic acid derivatives have been reported to be constituents of this plant. In our preliminary screening, the MeOH extract of the leaves from *G. robusta* showed significant cytotoxicity against human breast carcinoma (MCF-7), lung carcinoma (NCI-H460), and central nervous system carcinoma (SF-268) cell lines. This information encouraged us to further investigate the chemical constituents of the MeOH extract. Fractionation of the extract led to the isolation of forty-one compounds, consisting of three new compounds, graviquinone (**1**), *cis*-3-hydroxy-5-pentadecylcyclohexanone (**2**), and methyl 5-ethoxy-2-hydroxycinnamate (**3**), as well as thirty-eight known compounds (Figure 1). Herein, we discussed the isolation, structural elucidation, and cytotoxicity of these compounds.

## 2. Results and Discussion

Graviquinone (**1**) was isolated as a yellowish solid. The molecular formula was determined to be C_10_H_10_O_4_ on the basis of the molecular ion at *m/z* 194.0577 in the HR-EIMS spectrum. The ^1^H-NMR and COSY spectra showed the presence of symmetric *o*-protons at *δ* 6.13 (2H, d, *J* = 9.9 Hz, H-2 and -6) and 6.82 (2H, d, *J* = 9.9 Hz, H-3 and -5) together with an α,β-unsaturated carbonyl unit at *δ* 6.19 (1H, d, *J* = 15.7 Hz, H-8) and 6.63 (1H, d, *J* = 15.7 Hz, H-7). The ^13^C-NMR, HMQC, and HMBC spectra indicated that both the proton signals at *δ* 6.13 and 6.82 exhibited ^1^H-^13^C long range correlations with a carboxyl carbon at *δ* 183.6 (C-1) and a quaternary carbon at *δ* 68.1 (C-4). Moreover, H-7 and H-8 showed HMBC correlations with the quaternary carbon and a carboxylic carbon at *δ* 164.8 (C-9). Furthermore, this downfield-shifted quaternary carbon correlated with a hydroxyl group (*δ* 5.49, br s) to which it was attached. A methyl group at *δ* 3.64 was assigned as forming an ester bond with the carboxylic acid because of the presence of the HMBC correlation between the methyl proton and the carboxylic carbon instead of the quaternary carbon. Consequently, the structure graviquinone (**1**) was assigned to be 4-hydroxy-4-(3-methoxycarbonyl)ethenylcyclohexadien-1-one.

**Figure 2:**
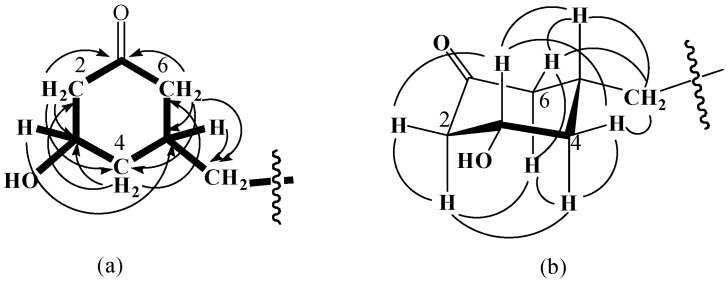
The key HMBC (a) and NOE (b) correlations of compound **2**.

Compound **2** was obtained as an optically active white oil. The HR-EIMS spectrum showed the molecular ion peak at *m/z* 324.3206, consistent with the molecular formula C_21_H_40_O_2_. The ^1^H- and COSY spectra demonstrate that a mutually coupled −CH_2_–CH–CH_2_–CH–CH_2_− unit is present, with a 15-carbon long chain at *δ* 0.88 (3H, t, *J* = 6.3 Hz, H-15'), 1.25 (24H, m, H-3'−14'), 1.28 (2H, m, H-2'), and 1.31-1.45 (m, H-1'). Methylenes at *δ* 2.33 (1H, t, *J* = 12.9 Hz, H-2_ax_) and 2.72 (1H, br d, *J* = 12.9 Hz, H-2_eq_) and then at 1.94 (1H, t, *J* = 13.2 Hz, H-6_ax_) and 2.37 (1H, br d, *J* = 13.2 Hz, H-6_eq_) exhibited HMBC correlations with a carbonyl carbon (*δ* 208.8), indicating the presence of a cyclohexanone ring (Figure 2a*δ* 3.91 (1H, m)] implied that a hydroxyl group was attached to C-3 (*δ* 69.2). The long chain substituent that was apparently attached to C-5 (*δ* 33.0) was assigned based on the HMBC correlations of H-5 [*δ* 1.60 (1H, m)] with C-1' (*δ* 36.5). The NOE correlations between H-3 and H-5 suggested the *cis* relative configuration of the two substituents in the cyclohexanone (Figure 2b). Therefore, compound **2** was assigned as *cis*-3-hydroxy-5-pentadecylcyclohexanone.

Compound **3** was determined to have the molecular formula C_12_H_14_O_4_ from the molecular ion peak at *m/z* 222.0891 in the HR-EIMS spectrum. The ^1^H-NMR signals at *δ* 3.81 (3H, s, 9-OCH_3_), 6.53 (1H, d, *J* = 16.0 Hz, H-8), 6.75 (1H, d, *J* = 8.6 Hz, H-3), 6.82 (1H, dd, *J* = 8.6, 2.5 Hz, H-4), 6.98 (1H, d, *J* = 2.5 Hz, H-6), and 7.96 (1H, d, *J* = 16.0 Hz, H-7) were very closely related to methyl 2,5-dihydroxycinnamate **4** [3], which implied that compound **3** could likewise be a 2,5-disubstituted cinnamic acid methyl ester derivative. The HMBC correlations between -OCH_3_, H-7, H-8 and C-9 (*δ* 168.1); H-7 and C-1 (*δ* 122.1), C-2 (*δ* 149.2), C-6 (*δ* 113.5), as well as the NOE correlation between H-6 and H-7, H-8, confirm this deduction. An ethoxy group at *δ* 1.39 (3H, t, *J* = 6.9 Hz) and 3.98 (2H, q, *J* = 6.9 Hz) was assigned as a substituent of C-5 owing to the signal at *δ* 3.98 (CH_2_). Moreover, this group demonstrated a HMBC correlation with C-5 (*δ* 152.9) and NOE correlations with H-4 and H-6. The hydroxyl group at *δ* 5.68 (1H, br s, 2-OH) appeared to be attached to C-2. Hence, the structure of **3** was determined to be methyl 5-ethoxy-2-hydroxycinnamate.

In addition to these three new compounds, thirty-eight known compounds were isolated from the MeOH extract of the leaves from *G. robust.* These known compounds could be divided into three types: benzenoid compounds, including methyl 2,5-dihydroxycinnamate (**4**), cinnamic acid, methyl coumarate, *p*-coumaric acid, pentacosyl dihydro-*p*-coumarate, methyl 3,4-dihydroxybenzoate, 4-hydroxybenzaldehyde, *p*-nitrophenol, methyl *p*-hydroxybenzoate, 4-hydroxyacetophenone, hydro- quinone, and *p*-hydroxybenzoic acid; alkylresorcinols, including gravicycle (**5**) [4], dehydrogravicycle (**6**) [4], robustol (**7**) [4], dehydrobustol-A (**8**) [4], dehydrobustol-B (**9**) [5], gravirobustol C (**10**) [5], methylgraviphane (**11**) [4], methyldehydrograviphane (**12**) [4], graviphane (**13**) [4], dehydrograviphane (**14**) [4], bisgravillol (**15**) [4], dehydrobisgravillol (**16**) [4], bis-norstriatol (**17**) [4], 5-[14'-(3'',5''-dihydroxyphenyl)-*cis*-tetradec-6'-en-1'-yl]benzene-1,3-diol (**18**) [4], gravirobustol A (**19**) [5], *cis*-5-*n*-pentadecylresorcinol (**20**) [4], and *cis*-5-*n*-pentadec-8'-enylresorcinol (**21**) [4]; and flavonoids, including rhamnocitrin [6], quercetin [7], kaempferol [8], rhamnetin [9], 7-*O*-methylrutin [10], eriodictyol-7-methyl ether [11], and sakranetin [11]. Two other compounds, grasshopperketone [12] and itaconic acid 4-methyl ester [13], were also identified by the comparison of their spectral data with those reported in the literature.

Compounds **1−8**, **11−18**, **20**, and **21** were subjected to cytotoxic evaluation. The clinically applied anticancer agent, actinomycin D, was used as a positive control for the cytotoxicity assays. Among these compounds, graviquinone (**1**) showed the strongest cytotoxicity against MCF-7, NCI-H460, and SF-268 cell lines, with IC_50_ values of 15.0, 10.8, and 5.9 μM, respectively (Table 1). *cis*-3-Hydroxy-5-pentadecyl-cyclohexanone (**2**) also showed moderate activity, with IC_50_ values of 26.7, 42.6, and 20.5 μM. The alkylresorcinols **5−8**, **11−18**, **20**, and **21** showed marginal cytotoxicity, with IC_50_ values of 39.8−22.8 μM [4]. The similar IC_50_ values of these compounds indicated that the alkyl chain structures, either cyclic or straight chain, had no impact on the anti-cancer activity of these alkylresorcinols. 

**Table 1:** Cytotoxicity of compounds **1–8**, **11–18**, **20**, and **21** toward some cancer lines.

Compounds	IC_50_ (μM) ^a^		
	MCF-7	NCI-H460	SF-268
**1**	15.0 ± 3.0	10.8 ± 2.3	5.9 ± 0.1
**2**	26.7 ± 1.9	42.6 ± 3.6	20.5 ± 0.6
**3**	>50	>50	>50
**4**	>50	>50	>50
Actinomycin D^b^	0.103	0.008	0.016

(a) Values were mean ± SD (n = 3–8). MCF-7 = human breast tumor cell line; NCI-H460 = human lung tumor cell line; SF-268 = human entral nervous system tumor cell line; (b) Positive control, IC_50_ values reported in [14].

**Figure 3:**
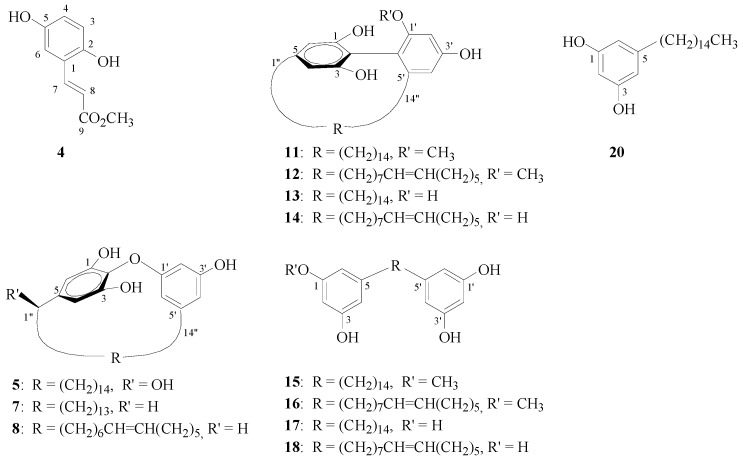
Structures of compounds **4**, **5**, **7**, **8**, **11–18**, and **20**.

Subsequently, compounds **1**, **4**, **5**, **7**, **8**, **11−18**, and **20** (Figure 3) were examined for their antioxidant properties based on the scavenging of the α,α-diphenyl-β-picrylhydrazyl free radical (DPPH) (Table 2). Amoung these compounds, methyl 2,5-dihydroxycinnamate (**4**), containing the *p*-dihydroxy functionality, together with graviphane (**13**) and dehydrograviphane (**14**), containing tetrahydroxycyclophane moieties, showed very potent activity, with IC_50_ values of 0.53, 3.96, and 2.05 μM, respectively. The results were compared with α-tocopherol, which is commonly used in the food industry as an antioxidant (IC50, 3.10 μM). The above compounds were also examined for their tyrosinase inhibition activities (Table 2). Methyl 2,5-dihydroxycinnamate (**4**) and bis-norstriatol (**17**) strongly inhibited L-DOPA oxidation by mushroom tyrosinase with IC_50_ values of 69.22 and 65.54 μM, respectively, and was compared with kojic acid (IC_50_, 114.54 μM) as a reference.

**Table 2:** DPPH inhibition activity and tyrosinase inhibition activity of compounds **1**, **4**, **5**, **7**, **8**, **11–18**, and **20**.

Compounds	IC_50_ (μM) or (Inhibition %) *^a^*	
	DPPH inhibition activity	Tyrosinase inhibition activity
**1**	23.01 ± 0.53	(32.78 ± 0.54)
**4**	0.53 ± 0.09	69.22 ± 1.27
**5**	25.68 ± 0.32	210.35 ± 2.13
**7**	28.78 ± 0.42	(30.87 ± 0.98)
**8**	11.78 ± 0.88	(37.92 ± 0.67)
**11**	(28.21 ± 0.23)	178.41 ± 2.23
**12**	15.67 ± 0.31	250.53 ± 3.26
**13**	3.96 ± 0.55	(23.66 ± 0.76)
**14**	2.05 ± 0.22	(42.76 ± 0.53)
**15**	36.73 ± 0.43	296.65 ± 3.32
**16**	28.93 ± 0.67	(48.22 ± 0.43)
**17**	31.31 ± 0.34	65.54 ± 0.87
**18**	35.22 ± 0.48	245.14 ± 2.21
**20**	(21.32 ± 0.21)	233.23 ± 1.02
α-tocopherol	3.10 ± 0.05	-
Kojic acid	-	114.54 ± 1.21

*^a^* Percentage of inhibition (Inh %) at the 16.7 g/mL concentration; Results are presented as mean ± S.E.M (n =3). Other compounds were not tested.

## 3. Experimental

### 3.1. General

Melting points were recorded on a Yanaco MP-3 melting point apparatus and were not corrected. Optical rotations were measured on a Jasco DIP-370 digital polarimeter. UV spectra were recorded on an Agilent 8453 spectrophotometer. IR spectra were recorded on a Nicolet Magna FT-IR spectrophotometer. NMR spectra were recorded on Bruker Avance 300 and AMX 400 FT-NMR spectrometers; all chemical shifts were given in ppm from tetramethylsilane as an internal standard. Mass spectra were obtained on a VG 70-250S spectrometer by a direct inlet system.

### 3.2. Plant Material

The leaves of *G. robusta* were collected on the campus of National Cheng Kung University, Tainan, Taiwan, in September of 2003. The samples were authenticated by Professor C.S. Kuoh of the Department of Life Sciences, National Cheng Kung University. A voucher specimen (No: PLW-0303) was deposited in the Herbarium of the same school.

### 3.3. Extraction and Isolation

The dry leaves of *G. robusta* (7.7 kg) were extracted with MeOH (6 × 10 L) for 6 h under reflux conditions. The filtrate was concentrated under reduced pressure to obtain a dark green syrup. This syrup was re-suspended in H_2_O (1.5 L) and then partitioned with *n*-hexane (6 × 1 L), CHCl_3_ (6 × 1 L) and EtOAc (6 × 1 L) to give *n*-hexane- (150 g), CHCl_3_- (15 g), EtOAc- (125 g) and H_2_O-soluble portions, respectively.

The *n*-hexane extract was subjected to silica gel column chromatography (CC), eluting with *n*-hexane−Me_2_CO (4:1) in a step gradient that gradually increased the polarity of the solution with pure Me_2_CO to afford eight fractions. Fractions 4–7 contained a large amount of resorcinols, which were difficult to separate. Purification of fraction 4 was accomplished using silica gel CC and eluting with a gradient of CHCl_3_-MeOH (50:1 to pure MeOH) to gave a mixture of methylgraviphane (**11**) and methyldehydrograviphane (**12**) (105 mg), a mixture of *cis*-5-*n*-pentadecylresorcinol (**20**), *cis*-5-*n*-pentadec-8'-enylresorcinol (**21**) (2.15 g), *cis*-3-hydroxy-5-pentadecylcyclohexanone (**2**) (3 mg), and pentacosyl dihydro-*p*-coumarate (12 mg), and a mixture of robustol (**7**), dehydrobustol-A (**8**), dehydrobustol-B (**9**), and gravirobustol C (**10**) (825 mg). Unfortunately, compounds **9**, **10** and **21** could not be obtained in pure samples. Fraction 5 was separated by using silica gel CC and by eluting with a gradient of CHCl_3_-MeOH (30:1 to pure MeOH) to give a mixture of bisgravillol (**15**) and dehydrobisgravillol (**16**) (35 mg) and a mixture of graviphane (**13**) and dehydrograviphane (**14**) (180 mg). Fraction 6 was separated by using silica gel CC and by eluting with a gradient of CHCl_3_-MeOH (20:1 to pure MeOH) to gave a mixture of bis-norstriatol (**17**), 5-[14'-(3'',5''-dihydroxyphenyl)-*cis*- tetradec-6'-en-1'-yl]benzene-1,3-diol (**18**), and gravirobustol A (**19**) (545 mg), which unfortunately could not be obtained as a pure compound. Using the same elution conditions as for fraction 6, fraction 7 produced a mixture of gravicycle (**5**) and dehydrogravicycle (**6**) (52 mg). However, extensive efforts were made through repeated CC to purify these components for identification.

The CHCl_3_ extract was subjected to silica gel CC, eluting with CHCl_3_-MeOH (20:1) in a step gradient that gradually increased the polarity with pure MeOH to afford five fractions. Fraction 2 was separated using silica gel CC, eluting with a gradient of (*i*-Pr)_2_O-MeOH (50:1 to pure MeOH), to give *p*-nitrophenol (6 mg), methyl *p*-hydroxybenzoate (4 mg), 4-hydroxybenzaldehyde (6 mg), cinnamic acid (1 mg), and graviquinone (**1**) (1.8 g), successively. Fraction 3 was purified by silica gel CC, eluting with (*i*-Pr)_2_O-MeOH (50:1 to pure MeOH), to obtain itaconic acid 4-methyl ester (2 mg), 4-hydroxyacetophenone (2 mg), methyl 3,4-dihydroxybenzoate (2 mg), and rhamnocitrin (4 mg). Fraction 4 was separated using silica gel CC, eluting with (*i*-Pr)_2_O-Me_2_CO (5:1 to pure Me_2_CO), to afford methyl coumarate (4 mg) and 5-ethoxy-2-hydroxycinnamate (**3**) (5 mg). Using the same elution conditions as for fraction 4, fraction 5 produced hydroquinone (2 mg), *p*-hydroxybenzoic acid (3 mg), and grasshopperketone (5 mg).

The EtOAc extract was subjected to silica gel CC, eluting with a gradient of CHCl_3_-MeOH (20:1 to pure MeOH) to yield twelve fractions. Fractions 1 and 2 were further purified by silica gel CC, eluting with a gradient of CHCl_3_-MeOH (6:1 to pure MeOH), to give quercetin (30 mg) and kaempferol (4 mg), respectively. Fraction 3 was separated using silica gel CC, eluting with CHCl_3_-Me_2_CO (10:1 to pure Me_2_CO), to afford methyl 2,5-dihydroxycinnamate (**4**) (1.1 g), eriodictyol-7-methyl ether (24 mg) and *p*-coumaric acid (18 mg). Fraction 5 was chromatographed using the same elution conditions as for fraction 3 to give sakranetin (10 mg), rhamnetin (18 mg), and 7-*O*-methylrutin (5 mg).

### 3.4. Cytotoxicity Assay

The cytotoxicity assay was carried out according to procedures that have been previously described in the literature [14].

### 3.5. Free Radical Scavenging Activity Assay

The free radical scavenging activity of compounds **1**, **4**, **5**, **7**, **8**, **11−18**, and **20** was measured with DPPH^•^ using the method of Chiu *et al*. [15]. Briefly, 0.1 mM solution of DPPH^•^ in ethanol was prepared, and sample (20 μL) was added. The sample was thoroughly mixed and kept in the dark for 30 min. The absorbance was measured at 517 nm on a Quant universal microplate spectrophotometer. α-Tocopherol (Sigma Chemical Co.) was used as a standard agent. A lower absorbance of the reaction mixture indicated a higher free radical scavenging activity. The sample concentration that was used to provide the IC_50_ data was calculated from a graph plotting inhibition percentage against sample concentration. Tests were performed in triplicate.

### 3.6. Tyrosinase Inhibitory Activity

The tyrosinase assay was carried out according to a procedure previously described in the literature [16]. The test substance was dissolved in 10% DMSO-H_2_O solution (0.1 mL) and then incubated with mushroom tyrosinase (0.1 mL, 135 U/mL, PBS pH 6.8) at 25 °C for 10 min. Next, L-dopa phosphate buffer solution (0.1 mL, 0.5 mM, pH 6.8) was added. The reaction mixture was incubated for 5 min. The amount of dopachrome in the mixture was determined by measuring the optical density (OD) at 475 nm using a Quant universal microplate spectrophotometer. Kojic acid (Sigma Chemical Co.) was used as a standard agent. The inhibitory percentage of tyrosinase was calculated as follows:

% inhibition = {[(*A* − *B*)/(*C* − *D*)]/(*A* − *B*)} × 100

Where:

*A*: OD at 475 nm without test substance;
*B*: OD at 475 nm without test substance and tyrosinase;
*C*: OD at 475 nm with test substance.

*Graviquinone* (**1**). Yellow amorphous powder. mp 79−80 °C. UV (MeOH) *λ_max_* (log ε) 213 (4.0), 372 (1.6) nm. IR (KBr) *ν_max_* 3423, 1715, 1664 cm^−1^. EIMS *m/z* (rel. int.) 194 (43, M^+^), 162 (100), 134 (83), 107 (36), 77 (33), 55 (49); HR-EIMS *m/z* 194.0577 [M]^+^ (calcd for C_10_H_10_O_4_ 194.0579). ^1^H-NMR (acetone-d_6_) *δ* 3.64 (3H, s, 9-OCH_3_), 5.49 (1H, br s, 4-OH), 6.13 (2H, d, *J* = 9.9 Hz, H-2 and -6), 6.19 (1H, d, *J* = 15.7 Hz, H-8), 6.63 (1H, d, *J* = 15.7 Hz, H-7), 6.82 (2H, d, *J* = 9.9 Hz, H-3 and -5); ^13^C NMR (acetone-d_6_) *δ* 50.2 (9-OCH_3_), 68.1 (C-4), 120.3 (C-8), 126.5 (C-2 and -6), 145.5 (C-7), 147.9 (C-3 and -5), 164.8 (C-9), 183.6 (C-1).

*cis-3-Hydroxy-5-pentadecylcyclohexanone* (**2**). White amorphous powder mp 62−64 °C [α]_D_ −101° (c 0.10, CHCl_3_) UV (CHCl_3_) *λ_max_* (log ε) 241 (3.1), 280 (2.9) nm IR (KBr) *ν_max_* 3424, 1709, 1595, 1469 cm^−1^ EIMS *m/z* (rel. int.) 324 (3, M^+^), 306 (6), 113 (100), 95 (19); HR-EIMS *m/z* 324.3206 [M]^+^ (calcd for C_21_H_40_O_2_ 324.3208). ^1^H NMR (CDCl_3_) *δ* 0.88 (3H, t, *J* = 6.3 Hz, H-15'), 1.25 (24H, m, H-3'−14'), 1.28 (2H, m, H-2'), 1.31–1.45 (3H, m, H-4_ax_ and H-1'), 1.60 (1H, m, H-5_ax_), 1.94 (1H, t, *J* = 13.2 Hz, H-6_ax_), 2.21 (1H, br d, *J* = 12.3 Hz, H-4_eq_), 2.33 (1H, t, *J *= 12.9 Hz, H-2_ax_), 2.37 (1H, br d, *J* = 13.2 Hz, H-6_eq_), 2.72 (1H, br d, *J* = 12.9 Hz, H-2_eq_), 3.91 (1H, m, H-3_ax_)H, H; ^13^C-NMR (CDCl_3_) *δ* 14.1 (C-15'), 22.7 (C-14'), 26.6 (C-2'), 29.3–29.7 (C-3'−12'), 31.9 (C-13'), 33.0 (C-5), 36.5 (C-1'), 41.0 (C-4), 47.0 (C-6), 50.9 (C-2), 69.2 (C-3), 208.8 (C-1).

*Methyl 5-ethoxy-2-hydroxycinnamate* (**3**). Yellow amorphous powder. mp 98−100 °C. UV (MeOH) *λ_max_* (log ε) 249 (3.65), 278 (3.77), 351 (3.41) nm. IR (KBr) *ν_max_* 3404, 1705, 1630, 1455 cm^−1^. EIMS *m/z* (rel. int.) 222 (8, M^+^), 162 (32), 149 (24), 134 (100), 107 (33), 105 (38), 77 (81); HR-EIMS *m/z* 222.0891 [M]^+^ (calcd for C_12_H_14_O_4_ 222.0892). ^1^H NMR (CDCl_3_) *δ* 1.39 (3H, t, *J* = 6.9 Hz, 5-OCH_2_CH_3_), 3.81 (3H, s, 9-OCH_3_), 3.98 (2H, q, *J* = 6.9 Hz, 5-OCH_2_CH_3_), 5.68 (1H, br s, 2-OH), 6.53 (1H, d, *J* = 16.0 Hz, H-8), 6.75 (1H, d, *J *= 8.6 Hz, H-3), 6.82 (1H, dd, *J* = 8.6, 2.5 Hz, H-4), 6.98 (1H, d, *J* = 2.5 Hz, H-6), 7.96 (1H, d, *J* = 16.0 Hz, H-7); ^13^C NMR (CDCl_3_) *δ* 14.9 (5-OCH_2_CH_3_), 51.7 (9-OCH_3_), 64.2 (5-OCH_2_CH_3_), 113.5 (C-6), 117.3 (C-3), 118.4 (C-8), 118.5 (C-4), 122.1 (C-1), 140.1 (C-7), 149.2 (C-2), 152.9 (C-5), 168.1 (C-9).

## 4. Conclusions

Three new compounds, graviquinone (**1**), *cis*-3-hydroxy-5-pentadecylcyclohexanone (**2**), and methyl 5-ethoxy-2-hydroxycinnamate (**3**), and thirty-eight known compounds were isolated from the leaves of *G. robusta*. Graviquinone (**1**) showed the strongest cytotoxicity against the tested tumor cell lines. In addition, our results also indicated that the alkyl chain structures, either cyclic or straight chain, had no impact on the anti-cancer activity of alkylresorcinols. Subsequently, methyl 2,5-dihydroxycinnamate (**4**) showed very potent DPPH inhibition activity with IC_50_ value of 0.53 μM, as compared with α-tocopherol. Moreover, methyl 2,5-dihydroxycinnamate (**4**) and bis-norstriatol (**17**) demonstrated strong inhibition activities for L-DOPA oxidation by a mushroom tyrosinase, with IC_50_ values of 69.22 and 65.54 μM, respectively.
